# Integrative Learning in US Undergraduate Public Health Education: A Review of Student Perceptions of Effective High-Impact Educational Practices at Georgia State University

**DOI:** 10.3389/fpubh.2019.00101

**Published:** 2019-04-30

**Authors:** Elizabeth Armstrong-Mensah, Kim Ramsey-White, Ernest Alema-Mensah

**Affiliations:** ^1^Department of Health Policy and Behavioral Science, Georgia State University, Atlanta, GA, United States; ^2^Morehouse School of Medicine, Atlanta, GA, United States

**Keywords:** integrative learning, high impact educational practices, undergraduate course work and assignments, service learning/community-based learning, collaborative projects, study abroad, undergraduate research, signature experience

## Abstract

In 2003, the United States (US) Institute of Medicine of the National Academies recommended that all undergraduate students have access to an education in public health to assist with diversifying the public health workforce and ensuring an educated citizenry on public health issues. In line with this recommendation, and that of the Consensus Conference on Undergraduate Public Health Education, Georgia State University established a Bachelor of Science in Public Health (BSPH) program in 2016, with the mission of advancing health through leadership, scholarship, research, and service, to better the human condition and to promote the common good, especially for urban communities in the US and for global populations. Using integrative approaches that encourage student empowerment, self-development, integrative thinking, and reflective learning, the Georgia State University BSPH program currently offers a range of generalist introductory public health courses to over 400 students. This review seeks to examine student perceptions of integrative practices utilized by Georgia State University faculty in the BSPH program and to investigate the extent to which student perceive these integrative educational practices as preparing them to use insights gained in the classroom and from the field, to question, modify, connect, and integrate material learned in the academic setting, to real-life public health challenges. It also seeks to identify which of the integrative educational practices have the highest impact of helping students integrate the knowledge and skills gained to public health issues.

## Introduction

Georgia State University is an urban public research institution located in Atlanta, Georgia. Established in 1913, the university has seven campuses throughout metro Atlanta with over 51,000 students of diverse backgrounds, enrolled in over 250 degree programs in 100 fields of study, including public health (1). In June 2016, The Council on Education for Public Health (CEPH) Board of Councilors accredited the School of Public Health (SPH) at Georgia State University. Georgia State University's SPH began as a Master of Public Health (MPH) program, accredited by CEPH since 2007—making it the first public university in Atlanta to gain that distinction (1). In 2016, a Bachelor's of Science in Public Health (BSPH) program was created within the SPH, with an emphasis on urban and global public health. The new BSPH program leverages the existing interdisciplinary make-up of Georgia State University's School of Public Health. The BSPH program seeks to prepare students for course work across public health disciplines, and to equip graduates with cross-professional competencies for public health jobs with urban, and global public health organizations. The SPH does this by utilizing integrative learning approaches that encourage student empowerment and self-development, and by equipping students with the requisite knowledge and skills to be integrative thinkers, critical and analytical problem solvers, and reflective learners. The establishment of the BSPH program supports Goal 3 of the Georgia State University Strategic Plan, as well as the recommendation of the Institute of Medicine (IOM) that, “all undergraduates should have access to education in public health” (2). The establishment of the BSPH program is also consistent with the IOM's call for expansion of undergraduate public health education to address two priority needs; (1) a serious disparity between the number of graduates produced by schools of public health and the number of workers needed (i.e., a workforce shortage), and (2) the need for a large, well-educated public health workforce that is able to respond effectively to emerging trends that impact population health, such as globalization, urbanization, population aging, health disparities, and alterations to the US health care system (3).

This review seeks to examine student perceptions of the high-impact educational practices (HIPs) utilized by Georgia State University faculty in the BSPH program, and the extent to which students see these HIPs as preparing them to use insights gained in the classroom and from the field, to question, modify, connect, and integrate academic material to real-life public health challenges. It also seeks to examine HIPs that have the greatest impact.

## HIPs and Student Learning

Students pursue a college education for several reasons, prime among them being the need for financial well-being and the ability to engage in leisure activities (4). As colleges and universities prepare students for their future careers, and work toward attracting the next set of potential students, they are faced with identifying educational practices that will prepare students for a dynamic and competitive workforce (5). These institutions are also faced with the question of whether their undergraduate students are academically engaged and learning enough in college, and whether their students are being taught foundational skills effectively (6).

Several studies have established a significant association between good educational practices and student college outcomes. Chickering and Gamsons assert that contact between students and faculty, reciprocity and cooperation among students, active learning, and prompt feedback are practices that can aid this process (7). These practices have been vetted, and found to positively influence undergraduate student learning and growth (8, 9) put together, these practices comprise high impact learning practices.

HIPs have been found to contribute to student increased academic engagement, knowledge, resolve, and general academic success (10). These educational practices afford students the opportunity to participate in activities beyond the classroom over a period of time, resulting in learning and personal development (11). HIPs not only enable students to apply what they have learned, or make meaning of their learning, they also contribute to metacognitive gains by students (12). In his article on HIPs, Kuh lists writing intensive courses, collaborative assignments and projects, undergraduate research, service learning, community-based learning, and <underline >diversity/global learning as practices that help to increase student retention and learning (13). According to Kuh, the implementation of HIPs among other things, has the tendency to increase student interaction with faculty and their peers on course work, and to induce reflection on course material and integrated learning (14). Engaging students in activities that focus on “learning by doing” has the tendency to make classroom learning real and relevant through the application of new knowledge to real life settings (15). Kinzie (16) noted that faculty engagement of students in projects in and outside the class increases student learning. Indeed project-based learning integrates knowing and doing. Through this effort, students not only learn elements of the core curriculum, but also apply what they know to solve authentic problems and produce results that matter (16). Students exposed to good educational practices tend to obtain better grades, and have increased cognitive, emotional, and personal growth. They also tend to be more satisfied with their college experience (17).

## Materials and Methods

### Setting and Population

The BSPH program has over 400 racially and economically diverse students enrolled in the public health major, and caters to both traditional and non-traditional students. The program is a 4-year degree that places special emphasis on urban and global health issues, and has a curriculum that focuses on elements of life and biological sciences, social sciences, and the humanities. The goal of the program is to provide students with an interdisciplinary understanding of public health, using a broad spectrum of approaches and course work. Students enrolled in the program acquire knowledge and skills needed for graduate school, and for careers in a wide range of public health and interdisciplinary professions.

### Sampling and Data Collection

We used a cross-sectional convenience sample of current and graduated BSPH students to assess student perceptions of the extent to which six HIPs implemented by Georgia State University's BSPH program faculty, impact undergraduate student cumulative learning, academic success, and career outcomes. Undergraduate course work and assignments, service learning/community-based learning, collaborative projects, study abroad, undergraduate research, and signature experience were the HIPs examined.

Students in the BSPH program were informed about the study and its purpose, through faculty announcements, emails, the BSPH general learning management system (iCollege), and were invited to take the survey. Completed survey questionnaires were collected on a daily basis using Qualtrics. All students associated with the program were given an equal opportunity to participate in the study.

To assess the impact of BSPH HIPs, data was collected on the amount of time students purposely devoted to course work and assignments, the extent of student interaction with peers and faculty on course work, and student opportunities to receive guidance and on-going feedback from faculty on their course work and assignments. Data was also collected on student's confidence in their ability to apply public health knowledge situations both in and outside the classroom.

The study was exempt from the Georgia State University IRB review process because, it was conducted in an established or commonly accepted educational setting that specifically involved normal educational practices that did not adversely affect students' opportunity to learn required educational content. The study was further exempt from IRB approval because it sought to research the effectiveness of or the comparison among instructional techniques, curricula, or classroom management methods.

### Variables and Measurement

The HIPs survey comprised a 23-item questionnaire containing closed-ended questions on a six-point Likert scale ranging from “strongly disagree” to “strongly agree.” The questionnaire was pilot-tested with randomly selected BSPH students for accuracy and consistency of measures. The questions assessed HIPs on six domains; (1) undergraduate course work and assignments, (2) service learning/community-based learning, (3) collaborative projects, (4) study abroad, (5) undergraduate research, and (6) signature experience. The survey questionnaire also asked for demographic and academic information.

The demographic and academic variables of the survey included academic status ranging from recent graduate to freshman, and the sex of students. The undergraduate course work and assignments variable focused on the amount of out of class time students devoted to course work and assignments, the availability of faculty to provide continuous feedback on course work and assignments, and student confidence in their ability to apply course material to real-life public health situations both in and outside the classroom. The service learning and community-based learning variable focused on career and occupational skills development, preparation for professional employment, and the application of classroom knowledge to service and community-based learning activities. The collaborative projects variable focused on working with peers, enhanced understanding of course material, and integrating and merging of knowledge from other courses with team members to produce a high quality academic product. The study abroad variable focused on decisions for postgraduate education and the acquisition of additional skills for a future career. The undergraduate research variable focused on student ability to research public health issues and their ability to present at conferences, and the final variable, signature experience, focused on the ability of students to create faculty reviewed academic products that were pertinent to their experience.

### Statistical Analysis

The data collected in Qualtrics were cleaned and exported to SPSS for analysis. Univariate analysis was conducted to summarize and describe data. Missing data were excluded from calculations. Univariate, bivariate, and multivariate data analyses were conducted using SAS 9.4 and Stata 11. Univariate analysis was conducted to obtain descriptive statistics for the six HIPs domains, sex, and educational status. Bivariate data analysis was conducted to determine the relationship between academic status, sex, and the six domains. At the multivariate level, we conducted an ordinal logistic regression to determine whether there was a relationship between academic status and the six HIPs domains and adjusted for sex. The 95% confidence interval was used to provide an estimated range of values for each of the HIPs variables measured.

## Results

### Univariate Statistics

#### Demographic and Academic Status

All 436 students currently enrolled in the BSPH program and the 19 students who have graduated from the program were invited to participate in the survey. One hundred and five students currently enrolled in the program and nine students who have graduated from the program, completed the survey. The majority of students (93.8%) are still enrolled in the program, and 88% of the respondents self-identified as female. Table 1 shows the academic level of the students who participated in the survey and Table 2 shows the number of students who responded to each of the HIPs domains by academic level.

**Table 1:** Academic level of students who participated in the survey.

**Academic level**	**Number**	**Percentage**
BSPH graduate	9	6.16
Freshman	2	1.37
Sophomore	17	11.64
Junior	40	27.40
Senior	46	31.51

**Table 2:** Number of students who responded to each of the high impact learning practices domain questions by academic level.

**HIPS domain**	**BSPH graduate *n* (%)**	**Freshman *n* (%)**	**Sophomore *n* (%)**	**Junior *n* (%)**	**Senior *n* (%)**	**Totals *n* (%)**
Undergraduate course work and assignments	8 (08.5)	2 (02.1)	15 (16.0)	31 (33.0)	38 (40.4)	94 (100)
Service learning/community-based learning,	9 (10.8)	2 (02.4)	13 (15.7)	25 (30.1)	34 (41.0)	83 (100)
Collaborative projects	9 (09.3)	2 (02.1)	14 (14.4)	34 (35.0)	38 (39.2)	97 (100)
Study abroad	3 (06.6)	1 (02.3)	6 (13.6)	12 (27.3)	22 (50.0)	44 (100)
Undergraduate research	7 (12.3)	1 (01.7)	7 (12.3)	13 (22.8)	29 (50.9)	57 (100)
Signature experience	9 (14.8)	1 (01.6)	8 (13.1)	14 (23.0)	29 (47.5)	51 (100)

#### Undergraduate Course Work and Assignments

Regarding undergraduate course work and assignments, 58% of students said they devoted a minimum of 3 h per day to their course work and assignments, 52% said they met with BSPH faculty outside of class for guidance on course work and assignments, and 66% said that they received feedback from BSPH faculty on their course work and assignments. Over half of the students surveyed (58%) said that they were confident that they could apply material from various courses offered by the program to public health issues both in and outside the classroom. Undergraduate BSPH courses include Health Equity and Disparities and Introduction to Public Health.

#### Service Learning/Community-Based Learning

Forty-eight percentage of students in the BSPH program said service learning and community-based learning was an important part of their BSPH experience, and 49% said the experience helped them to develop career and occupational skills. Fifty-two percentage of students indicated that they were able to apply their classroom knowledge to the service and community-based activities they engaged in. Fifty-six and 54% of students, respectively, said that their participation in service and community-based activities gave them a sense of personal achievement and prepared them for professional employment opportunities. Forty-seven percentage of the students surveyed disclosed that they were able to integrate their classroom work into their service and community-based activities.

#### Collaborative Projects

On the issue of collaborative projects, 45% of students stated that working with peers was an important part of their BSPH experience, while 8% did not think so. Forty percentage of students stated that collaborative projects helped them to better understand course material. Less than half of the students surveyed (40%) said group projects inspired them to do their best on assignments, and 42% of students indicated that group projects allowed them to integrate the ideas and knowledge they acquired from various program courses, with that of their peers to create good academic products (papers, projects, and presentations).

#### Study Abroad

A little more than a third (36%) of students reported that their study abroad experience motivated them to consider pursuing graduate studies, and allowed them to acquire additional skill sets for their future careers. Less than half (39%) of the students neither agreed nor disagreed that it influenced their decision to enter the public health workforce or pursue graduate education.

#### Undergraduate Research

Undergraduate research is one of the most requested opportunities from the student body. Forty-two percentage of the respondents took a course that required them to do some research. As a result, 43% of these students were able to use their research skills to develop oral and poster presentations delivered both on and off Georgia State University's campus.

#### Signature Experience

Given that the BSPH program is only in its third year of development, and the signature experience courses are taken when the predominance of the major coursework is completed, most of the respondents in the program have not taken the signature experience course yet. Of those who have, 42% stated that they were able to integrate and apply what they had learned in the classroom to their signature experience, and 42% said that enrolling in the course exposed them to experiences they would otherwise not have gained in the classroom. Forty-two percentage of students who took the signature experience course indicated that, with guidance from faculty members, they were able to produce good final academic products.

#### Bivariate and Multivariate Analysis

We conducted bivariate analysis for sex, academic status (including BSPH graduates, seniors and juniors) by all the six HIPs domains. We found no statistically significant relationship between sex and the six HIPs domains, but we found statistically significant relationships between BSPH graduates and juniors for collaborative projects (*p* = 0.0357) and signature experience (*p* = 0.0085) with regards to the question, *I am/was able to apply what I learned in the classroom to my signature experience*. When it came to BSPH graduates and seniors, we also found statistically significant relationships for collaborative projects (*p* = 0.0456) regarding the question, *Working on group projects collaboratively helps/helped me to integrate my ideas with those of others in the group to produce a good product*; for study abroad (*p* = 0.0331) with regards to the question, *Participating in the BSPH study abroad program allowed me to acquire additional skill sets for my future career and signature experience;* and for signature experience (*p* = 0.0379) in relation to the question*, I was able to apply my undergraduate research skills by presenting at a conference*. Concerning seniors and juniors, we found statistically significant relationships for undergraduate course work and assignments (*p* = 0.0065) regarding the question, *Faculty in the BSPH program provide/provided feedback on my coursework and assignments*; for service learning/community-based learning (*p* = 0.0464) with respect to the questions *The BSPH service-learning experience will help/helped with the development of career/occupational skills* and *I am/was able to apply classroom knowledge to my service learning activities;* and for collaborative projects (*p* = 0.0324) concerning the questions, *Collaborative group projects helps /have helped me to better understand course material and “Working collaboratively on group projects inspires /inspired me to do my best in an assignment., undergraduate research*. Concerning seniors and juniors, we also found statistically significant relationships for undergraduate research (*p* = 0.0224) regarding the question, *I participated/was able to participate in undergraduate research opportunities as a requirement of a class that I took in the BSPH program*; and for signature experience (*p* = 0.0448) in relation to the question, *The BSPH signature experience caused me to produce a final product that was evaluated by a faculty member*.

To examine the relationship between student educational levels in the BSPH program and the six HIPs domains, we performed multivariate analysis and adjusted for sex (using Stata 11).

Table 3 shows the overall outcomes from the ordinal logistic regression model for each of the six HIPs domains. Statistically significant relationships were found between all of the HIP domains and student academic level. Service learning/community-based learning had the strongest impact in our ordinal logistic model with an odds ratio of 7.117. This domain had more than seven times impact, compared with the other domains, which were all protective (having odds ratios <1). It is therefore likely that more students benefitted from the service learning/community-based learning than the other HIPs domains.

**Table 3:** Ordinal logistic regression model of respondents' domain by educational levels—adjusting for sex.

**Domains**	**Adjusted OR (95% CI)**		***p*-value**	**Overall *p*-value**
Undergraduate course work and assignments	0.057	(0.004–0.825)	0.036	0.0009
Service learning community-based learning	7.117	(1.423–35.58)	0.017	0.0493
Collaborative projects	0.531	(0.0344–8.187)	0.650	0.0109
Study abroad	0.223	(0.0187–2.666)	0.236	0.0093
Undergraduate research	0.0266	(0.0010–0.681)	0.028	0.0015
Signature experience	0.486	(0.241–0.976)	0.042	0.0203

## Discussion

Results from the study provide baseline data of the effectiveness of HIPs utilized by the BSPH faculty to educate and prepare students for real-life public health challenges. The results also indicate that students perceive the HIPs implemented in the program as effective in impacting their learning and preparing them for real-life public health challenges.

### Undergraduate Course Work and Assignments

Our study revealed that most students in the BSPH program (58%) devote more than 3 h per week per semester on undergraduate course work and assignments. This is consistent with the National Survey of Student Engagement's (NSSE) findings, that the average student spends about 17 h per week on course work including homework, reading, and assignments. For the majority of students responding to the survey, interaction with faculty outside classes provided additional guidance on how to do their coursework and assignments. Previous research indicates that student-faculty interactions can have several positive influences. Indeed, Pascarella and Terenzini found that student-faculty interaction generally have a positive influence on the cognitive growth and development of college students (9). They also found that student-faculty interaction is positively related to students' academic achievement and that the frequency of contact is related to students' positive learning outcomes (9).

### Service Learning/Community-Based Projects

Service learning affords students the opportunity to apply knowledge gained in the classroom to practical community-based projects. It also allows students to make real-life connections between what they learn in the classroom and what actually happens in practice. Close to half (48%) of the students who responded to our survey found service learning/community-based learning to be beneficial. This statistic is modest and may be because of the infancy of the BSPH program and the fact that not many students are at a point where they can engage in the practice. We found that participating in service learning activities was a catalyst to student development of skills for future employment, increased personal insight, cognitive and social development, and created a sense of personal and academic achievement. This is consistent with earlier research findings by Austin et al. (18), Yoiro et al. (19), and Conway et al. (20).

### Collaborative Undergraduate Projects

The goal of collaborative undergraduate projects is to help students learn to work and solve problems in the company of others, and to sharpen their understanding by listening seriously to the insights of others, especially those with different backgrounds and life experiences. Unfortunately, not as many BSPH students (40%) found this HIP to be helpful in increasing their understanding of course material. These results are consistent with the findings of a previous study conducted by Premo et al. which found that, collaborative projects by themselves are not enough to promote increased student achievement (21). Group projects sometimes end up becoming a source of friction between students. At their best, collaborative projects foster productive team and idea sharing among future professionals, while at their worst, team projects force high-achieving students to compensate for those less willing to put in the effort. It may be because of the latter reason that this HIP was not as popular with BSPH students who responded to our survey (22).

### Study Abroad

Brazil, The Dominican Republic, China, India, and Uganda are the five countries where students in the BSPH program have had the opportunity to visit and explore different cultures, worldviews, and life experiences. Led by full-time faculty from the SPH, as well as faculty from geosciences, criminal justice and communications, students who took the survey indicated that studying abroad enabled them to acquire additional skills sets such as critical thinking, problem solving, communication, leadership, professionalism, and intercultural fluency that they need for their future careers. These competencies are consistent with the National Association of College and Employers (NACE) key career competencies (23). All BSPH programs are designed to prepare students to be informed and exposed to public health issues from an urban and global perspective. Through mentoring, a challenging overseas academic program, and hands on experiential activities, students are positioned to gain invaluable knowledge and skills that augment their academic preparation. Consistent with Sanchez's (24) previous research finding, we found that the study abroad experience by itself is an example of several HIPs wrapped into one academic experience that lasts for a lifetime, transforms student intellectual perspectives and personal growth, and causes students to rethink their majors and incorporate further studies abroad into their academic schedules (24).

### Undergraduate Research

Even though the number of students who indicated that they were able to participate in undergraduate research was <50% of those who responded, 43% reported that their understanding of how to conduct research, increased their ability to write papers and to present at conferences, both on and off campus. This is consistent with Lopatto's (25) findings on undergraduate research and HIPs. He found that the undergraduate research experience affected student career plans and sometimes helped them to fine-tune their career plans (25). We found evidence of a connection between undergraduate research experiences, and personal academic growth and confidence in foundational research methods, and increased interest in graduate education and preparation for the labor force. Lin et al. in a previous study confirm that working with faculty is an important component of a successful undergraduate research experience (26).

There is no other extracurricular activity that undergraduate students at Georgia State University request more, than doing research with faculty. Students focused on continuing their education beyond the baccalaureate degree, know that learning how to conduct public health research with experienced faculty can enhance their graduate school applications considerably. Thus, in the 3 years that the BSPH program has been in operation, opportunities for students to participate in undergraduate research has continued to increase. In the spring of 2017, just one semester after the program was inaugurated, a faculty member agreed to work with two undergraduate students on a funded research project to examine the impact of mindfulness on tobacco cessation in low-income communities. Since that spring, in each semester, additional faculty have worked with more and more undergraduate students in the program. As of the fall 2018 semester, four faculty members are working with more than 10 students on research projects related to health policy, water quality, nano-particles, and health promotion and behavior. Additionally, the lead author of this paper has started a research club to work with students to develop manuscripts based on primary and secondary data analysis and the systematic reviews of the literature. Within a week of the faculty member announcing the launching of the club, over 20 students had signed up.

### Signature Experience

All students at Georgia State University are encouraged to complete at least one signature experience. Utilizing experiential strategies, signature experience courses give students the opportunity to apply course content to real-life situations. While signature experience courses are highly encouraged across the university, they are not a required course in any program of study except in the BSPH program. In this program, students entering their senior year of study register for PH 4991 (Signature Experience 1–Prospectus) and PH 4992 (Signature Experience–Capstone). Both courses are offered every semester in a 7-week mini-mester, so both courses can be taken in one semester. This increases the odds for students to progress to graduation in any given semester. The prospectus course (4991) is the first of a two-course sequence required to meet the area H requirements of the BSPH program of study. Students have the opportunity to integrate, synthesize and apply their public health knowledge through cumulative and experiential activities. In this course, students complete a variety of projects, and written assignments designed to assess student acquisition of the required public health competencies covered within the public health major. Mastery of these competencies is required to receive the BSPH degree.

Forty-two percentage of students indicated that participation in the signature experience course exposed them to things that typically do not occur in a traditional classroom, and that they were able to integrate what they learned in the classroom into the course, and were able to produce quality, culminating artifacts that were evaluated by their faculty. This finding is consistent with that of Fitzpatrick et al. (27) who assessed undergraduate public health capstone courses in 2016. In their study, they found that capstone projects caused students to integrate what they had learned from various courses, into their capstone experience and that, their success was most evident in their ability to transform draft literature reviews into a final product (27).

## Limitations

While the results from the assessment are promising, it would have been good to have more BSPH graduates complete the survey. To address this challenge, faculty will ensure to create awareness among BSPH students of the importance of participating in such surveys in the future.

## Conclusion

The study assessed six HIPs utilized by Georgia State University BSPH faculty and the extent to which integrative educational practices prepare students for the labor force and for further education. It also sought to identify integrative educational practices that have the highest impact of helping students integrate knowledge and skills gained in the classroom to public health issues. Results from the study provide invaluable baseline data for the assessment of future integrative educational HIPs utilized by Georgia State University faculty in the BSPH program.

## Author Contributions

EAr-M and KR-W wrote the draft manuscript, finalized, and edited the final manuscript. EAl-M performed the statistical analysis. All authors reviewed and approved the final manuscript.

### Conflict of Interest Statement

The authors declare that the research was conducted in the absence of any commercial or financial relationships that could be construed as a potential conflict of interest.
